# The Effect of Textile Structure Reinforcement on Polymer Composite Material Mechanical Behavior

**DOI:** 10.3390/polym16243478

**Published:** 2024-12-13

**Authors:** Svetlana Risteska, Vineta Srebrenkoska, Silvana Zhezhova, Sara Srebrenkoska, Sanja Risteski, Sonja Jordeva, Saska Golomeova Longurova

**Affiliations:** 1 Faculty of Technology, Goce Delcev University, Krste Misirkov 10-A, P.O. Box 201, 2000 Shtip, North Macedonia; svetlana.risteska@ugd.edu.mk (S.R.); silvana.zezova@ugd.edu.mk (S.Z.); sanja.risteski@ugd.edu.mk (S.R.); sonja.jordeva@ugd.edu.mk (S.J.); saska.golomeova@ugd.edu.mk (S.G.L.); 2 Faculty of Mechanical Engineering, Goce Delcev University, Krste Misirkov 10-A, P.O. Box 201, 2000 Shtip, North Macedonia; sara.srebrenkoska@ugd.edu.mk

**Keywords:** textile structure, polymer composite, thermal analysis, mechanical properties

## Abstract

Investigating the impact of textile structure reinforcement on the mechanical characteristics of polymer composites produced by the compression molding technique was the goal of this work. An epoxy resin system served as the matrix, and various woven (plain, twill, basket), nonwoven (mat), and unidirectional (UD) textile structures made from E-glass fibers were employed as reinforcement elements. Compression molding of pre-impregnated textile materials (prepregs) was used to create the composites. The well-impregnated textile structures with resin into prepreg and the good interface between layers of the composites were verified during the manufacture of the polymer–textile composites using DSC thermal analysis and an SEM microscope. For the mechanical behavior, flexural properties were determined. The composite samples with unidirectional prepreg reinforcement have the highest longitudinal flexural strengths at roughly 900 MPa. The woven prepreg-based composite laminates show balanced flexural properties in both directions. Composites based on plane and basket prepregs have a flexural strength of about 450 MPa. Their flexural strength is over 20% lower than that of the samples made using twill prepreg. In both directions, nonwoven prepreg-reinforced composite samples show the least amount of resistance to bending stresses (flexural strength of roughly 150 MPa).

## 1. Introduction

The textile reinforcement in polymer composite materials plays a crucial role in determining the mechanical behavior of the composite. The structure, type, and orientation of the textile reinforcement can significantly influence properties such as strength, stiffness, toughness, fatigue resistance, and failure mechanisms. Below is an in-depth exploration of how different textile reinforcements impact the mechanical behavior of polymer composites [1,2,3,4,5,6,7,8]. Textile reinforcements for polymer composites typically come in several forms, including woven fabrics (comprising two sets of yarns—warp and weft—interlaced at right angles), knitted fabrics (made by interlocking yarns in a loop formation, which provides flexibility), nonwoven fabrics (these are created by bonding fibers together without weaving or knitting), and braided fabrics (yarns are interlaced in a braid-like structure, providing good through-thickness properties). As a result of their specific geometry and characteristics, they have different behaviors and can be used in various applications [9,10,11,12,13]. Each type of textile reinforcement offers distinct benefits and compromises: woven fabrics are typically chosen for their high stiffness, knitted fabrics for their flexibility, nonwoven fabrics for their impact resistance, and braided fabrics for their superior strength through thickness.

With the advancement of technology, textile composites are increasingly used in structures subjected to a wide spectrum of static loads, including low/high-velocity impacts, during their lifetime [14] to replace not only metals but also their lightweight alloys. Textile composites are fabricated by introducing reinforcing fibers, in a woven or nonwoven form, into thermosetting or thermoplastic matrix materials to endow them with structural rigidity and stability [14]. The fiber material, reinforcement architecture, weaving pattern, fiber orientation (straightness/deviation from a straight path), stacking sequence, and number of fabric layers are important factors to be considered in designing textile composites [15]. Textile composites can be monolithic (only one type of fiber material is used) or hybrid (more than one fiber material). Morye et al. [16] experimentally investigated the effect of various matrix and reinforcement materials on the mechanical properties of various textile composites with Nylon 66 and aramid fibers and phenol formaldehyde and polyvinyl butyral matrices. Wong et al. [17] estimated the delamination area and compression strength for five textile composites with E-glass fiber plies and different matrix materials. Isa et al. [18] investigated the mechanical properties of monolithic Kevlar, glass- and nylon-reinforced textile composites and hybrid Kevlar/glass, nylon/glass, and Kevlar/glass/nylon textile composites with an unsaturated polyester resin matrix.

Textile composites based on unidirectional reinforcement possess much better in-plane mechanical properties but are prone to delamination. To cope with this drawback, woven/braided/knitted fabric composites [19] are used to achieve better mechanical properties in both in-plane and transverse directions [20]. For laminated composites, the first-order shear deformation theory and semiempirical formulae have been used in multiple studies [21,22,23] to analyze their mechanical behavior by predicting such process parameters as the peak force, contact duration, peak strain on the back surface, etc. Various tools [20], such as the rule of mixtures, theory of random functions, boundary variation methods, composite cylinder models [24], and finite element analysis [25,26], have been employed for estimating the mechanical response of textile composites. Along with fabric properties, matrix properties and composite processing techniques also govern the failure mechanisms of woven textile composites [27,28,29,30,31]. In woven composites, warp and weft yarns are interlaced in a definite sequence, and specific fiber–matrix volume ratios and lamination configurations are used to produce materials with a higher transverse tensile strength, fracture toughness, and dimensional stability than UD composites [32]. Naik et al. [33] analyzed the failure mechanism in different plain-weave laminated composites made from glass/epoxy and T300/5208 carbon/epoxy and subjected to low-velocity transverse impact loads causing delamination due to interlaminar or in-plane stresses. Woven fabrics made of high-performance fibers, such as aramid (Kevlar/Twaron), UHMWPE (Spectra, Dyneema), PBO (Zylon), and AuTx, are effective materials for protection against impacts, but woven fabrics made of high-stiffness fibers such as carbon and glass are excellent for structures subjected to high compressive loads [34,35]. Textile composites are now increasingly employed in fighter and commercial aircraft, trains, racing cars, etc.

The mechanical strength of textile-reinforced polymer composites is primarily determined by fiber properties (e.g., tensile strength, fiber diameter) and the fiber–matrix interaction. The textile reinforcement orientation plays a key role: unidirectional (UD) fabrics have fibers oriented in one direction, providing maximum strength in that direction; however, the composite will be weak in the transverse direction. Woven textiles offer strength in both directions (warp and weft), but their strength is generally lower than that of unidirectional textiles because of the inherent crimp (bending) in the fibers due to the weaving process. Braided textiles provide balanced strength in multiple directions, making them suitable for applications requiring isotropic strength characteristics (equal strength in all directions). Stiffness (measured by the modulus of elasticity) is influenced by the fiber orientation and the volume fraction of reinforcement. Unidirectional fibers typically result in higher stiffness in the fiber direction. However, woven fabrics can offer more balanced stiffness in multiple directions, making them more isotropic but typically less stiff compared to unidirectional composites [14,15,16,17,18,19,20]. In composite materials, fatigue failure often occurs due to repeated cyclic loading, which leads to matrix cracking and delamination. Textile reinforcements can influence the fatigue resistance of the composite. Woven and braided fabrics can help delay fatigue failure because they provide more resistance to crack propagation due to the interlocking nature of the fibers, so unidirectional fabrics are more susceptible to fatigue damage in the transverse direction since the matrix is more likely to experience delamination when subjected to cyclic loads [16,17,18,19,20,21,22]. The development of textile composites, their design, and manufacturing technologies is one of the most important achievements in the engineering of materials [14]. The outstanding achievements in the field of computer-aided design and manufacturing have facilitated the adaptation of many traditional textile processes to create 2-D and 3-D textile structures at low production costs. Textile composites have numerous applications across various industries, such as the aerospace industry, the automotive industry, railways, the marine industry, commercial mechanical engineering applications, civil engineering, buildings, protective and sports equipment, etc. This is due to the outstanding physical, thermal, and favorable mechanical properties, particularly lightweight, high stiffness and strength, good fatigue resistance, excellent corrosion resistance, and dimensional stability [16,17,18,19,20,21,22,23,24,25,26,27,28,29,30,31,32,33]. In their paper, Chowdhury et al. [34] provide a review and detailed information for different woven and nonwoven textile structures; they also provide discussions, including the fabrication processes, the relationship between fabric structure and composite properties, and morphological characteristics encompassing the current state of the art in woven fabrics for composite reinforcement.

In many final composite products for different applications, fabrics are first produced in the form of prepregs for better resin control and better impregnation-soaking of the fibers in order to have better mechanical characteristics. Pre-impregnated semi-finished textile products (prepregs) are a type of composite material that combines reinforcement fibers (such as glass, carbon, or aramid) with a resin matrix (usually epoxy, polyester, or phenolic) that has already been impregnated into the fibers. The term “prepreg” is short for “pre-impregnated”, indicating that the fibers have already been impregnated with resin before being used in manufacturing composite structures. Glass prepregs are used in composite materials for their excellent mechanical properties, ease of processing, and versatility in various industries. They are particularly valued in applications where high strength, durability, lightweight, and precision are critical, including aerospace, automotive, wind energy, and sporting goods, among others [35,36,37,38,39,40,41].

A state-of-the-art area in materials science and engineering, polymer–textile composites combine the remarkable qualities of textiles and polymers to produce high-performance, multipurpose materials. Depending on the intended usage, different textile structure reinforcements can be employed to make polymer composites. The production of polymer–textile composites involves a number of pieces of machinery, such as knitting, weaving, and melt extrusion machines. With the use of these tools, the structure and content of the composites may be precisely controlled, producing materials with unique qualities for particular uses [24,25,26,27,28,29,30]. In many applications, polymer–textile composites can be utilized to replace current metal and non-metal components, and tooling costs are comparatively low when compared to metal assemblies. Their characteristics make them perfect for use in electronics; for example, they can be widely employed in the production of circuit boards, televisions, radios, computers, cell phones, electrical motor covers, and more. Additionally, they have a wide range of uses in aircraft and aviation, usually in bulkheads, ducting, storage bins, antenna enclosures, luggage racks, instrument enclosures, and engine cowlings. Glass textile-reinforced polymer composites play a crucial role in the manufacturing of wind turbine blades, offering high strength and flexibility while keeping weight to a minimum, which is essential for the efficiency and performance of wind turbines. They are widely suited for medical applications due to their hard-wearing finish, low porosity, and non-staining properties. Glass textile–polymer composites can be used for everything from instrument enclosures to X-ray beds, where X-ray transparency is crucial. Additionally, they can be widely applied to car components such as engine covers, bumpers, door panels, seat cover plates, and body panels.

This paper presents the influence of different textile preforms on the mechanical behavior of E-glass/epoxy composite plates. For that purpose, different types of textile structures from E-glass fibers were used as reinforcing components in the composite materials. From all textile preforms, prepregs with suitable characteristics were produced and subsequently processed into composite plates by using compression molding technology. The flexural strength and stiffness of manufactured samples were determined by using a three-point bending method. The resin content and baking method were the same for all products with different textile structures. The resulting composite structures meet the requirements for modern advanced materials that simultaneously provide excellent strength and low weight and that can be used as load-bearing structural parts in various industries. The results will assist in the selection of the textile preforms, which is an important prerequisite for the design and production of new high-performance textile-based composites for a wide range of applications. This paper summarizes the possibilities of using textile reinforcement in polymer composites for flexural strengthening. Compared to conventional steel reinforcement, cloth reinforcement in composites is substantially lighter. This makes it possible to reduce the construction’s weight, which lowers the total amount of materials used. Textile reinforcements made of glass fibers are very resistant to environmental degradation. The lifespan of constructions is increased by the fact that textile materials do not rust or degrade over time like standard steel reinforcement does [33,34,35,36,37,38,39,40,41].

## 2. Materials and Methods

### 2.1. Materials

In this study, different textile structures (unidirectional, woven, and nonwoven) from E-glass fibers as a reinforcement component and epoxy resin system as a matrix were used for the production of the polymer composites. Three types of woven fabrics with different weave patterns, i.e., plain, twill, and basket, and a nonwoven (mat) material were used with an areal mass for all of approximately 325 ± 15 g/m^2^ and thickness of around 0.32 ± 0.05 mm. The physical characteristics and assignation of the textile structures (woven and nonwoven) are summarized in Table 1, and the characteristics of components of the epoxy resin system are presented in Table 2.

**Figure 1:**
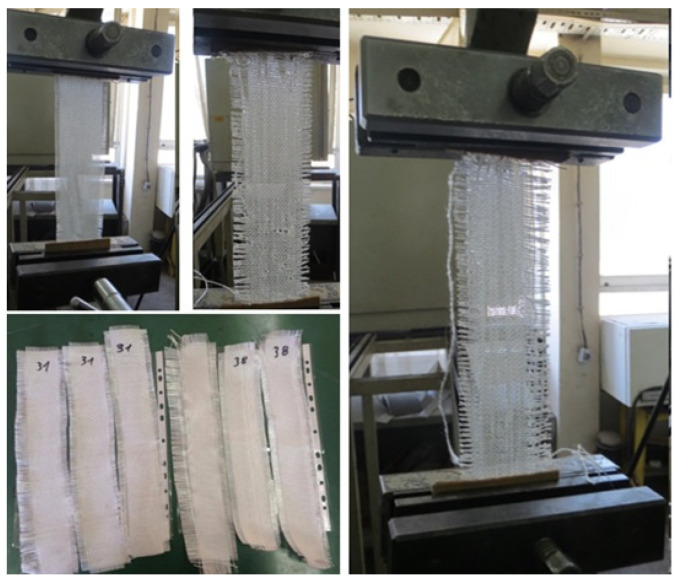
Tensile strength test using the universal test machine by Schenck.

The structural characteristics of woven fabrics have a significant influence on the appearance, durability, and behavior of woven fabrics in different applications. They are mostly determined by the type of yarn, as well as by the process parameters of the weaving machine. The key structural characteristics of woven fabrics are the type of weave structure, yarn type and count, yarn density, fabric weight and thickness, etc. [34,36]. Electron microphotographs of the structure of the used E-glass woven fabrics were obtained using a binocular microscope ZOOM 645 and a scanning electron microscope type VEGA3 LMU from the Tescan company, Brno–Kohoutovice, Czech Republic. These photos were taken to observe the differences in the weave structure of the applied woven fabrics, that is, the way of interweaving the weft and warp yarns. Additionally, tensile strength and elongation at break in the longitudinal and transverse directions were determined according to the ASTM D 5035 standard [42]. A Schenck Universal Tensile Strength Testing Machine was used for this test. The tensile strength of woven fabrics made of the most important mechanical properties makes them superior for many industrial applications compared to knitted and nonwoven textile materials. For this purpose, from all three types of woven fabrics, three samples (strips) with dimensions of 200 × 48 mm were cut in the weft and warp directions. The samples were fixed in the test machine with the help of 60 mm wide clamps (Figure 1).

**Figure 2:**
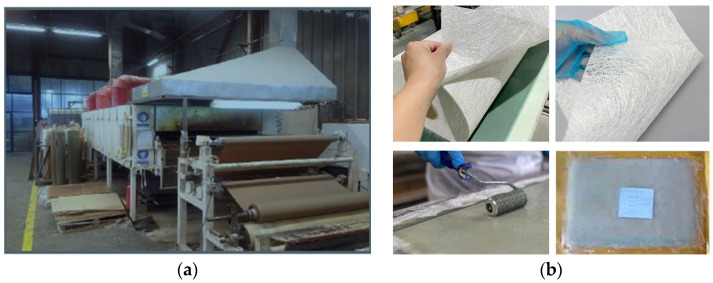
(**a**) Machine impregnation. (**b**) Hand impregnation.

From all woven fabrics, prepregs with suitable characteristics were produced in the laboratories of the company Laminati Kom D.O.O. Prilep, R. North Macedonia by using the impregnation machine (Figure 2a). Nonwoven textile structures were hand-impregnated into prepregs (Figure 2b), and the UD glass prepreg was the commercial product SIGRAPREG^®^ from SGL company, Wiesbaden, Germany. Making a good prepreg requires precise control and a good understanding of the resin flow and how it behaves under the influence of the heat or pressure being added in the cured state [24]. The basic characteristics for all produced prepregs are summarized in Table 3, and in the same table, the characteristics of the commercial UD prepreg are presented.

### 2.2. Preparation of Composite Plates

**Figure 3:**
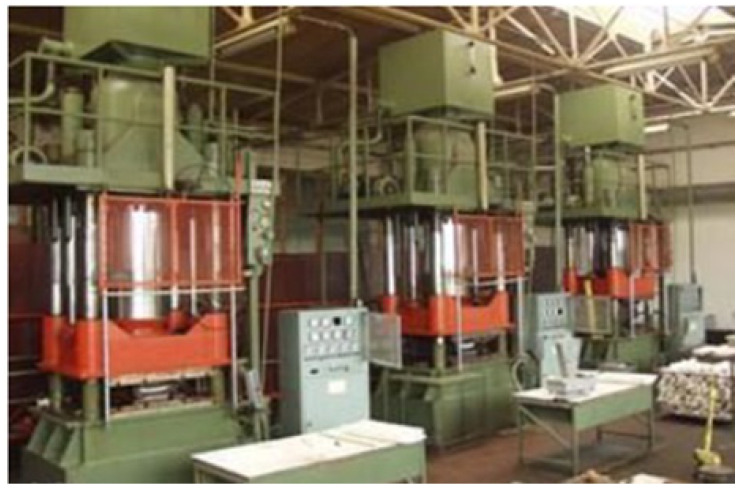
Press machine.

From all five different prepregs, laminate panels (composite plates) were produced by using compression molding technology. The samples were produced in the laboratories at the company Laminati Kom d.o.o. in Prilep. Laminate panels with dimensions of 250 mm × 200 mm were produced by stacking 10 prepreg layers from all five different types, wrapped with fireproof paper, and carefully placed in the open mold press machines (Figure 3). The laminates were performed with a specific pressure (from the machine) of 14 kg/cm^2^ (30 bar) for all five samples. However, the compression temperature for the samples from the produced prepregs (I, II, III, and IV) was 80 °C for a curing of 1 h and 110 °C for a post-curing of 1 h. To produce laminate samples from the commercial UD prepreg, the compression temperature was 90 °C for a curing of 1 h and 120 °C for a post-curing of 1 h, based on the recommendation from its manufacturer (Table 4). After the press cycle, the composite plates were left to stand for a few hours to allow the resin mixture to fully combine and dry out, ensuring complete curing. Then, from all five specimens (composite plates), the test specimens were cut in the MD and CD directions according to the testing standard for mechanical characterization.

### 2.3. Content of the Constituents and Voids in the Manufacture Plates

The content of the constituents in the manufacture plates was determined according to the ASTM D3171 standard [36], while the content of voids was tested following the ASTM D792 and ASTM D2734 standards [37,38]. Determining the content of the constituent’s components is significant from the aspect of modeling the material properties (mechanical, physical, thermal) of the composite structure, which depends on the reinforcing component and the matrix. Assessing the constituent content is crucial for evaluating the quality of the fabricated material. This helps ensure that the processes used during fabrication produce materials that meet the desired standards. The percentage of voids in the material is an important factor, as high void contents can negatively affect mechanical properties. High void contents often lead to lower fatigue resistance, greater susceptibility to moisture and atmospheric influences, and increased variation in strength [43,44,45,46]. Therefore, measuring void content serves as a critical quality indicator for composite materials.

### 2.4. Mechanical Characterization of the Manufacture Plates

**Figure 4:**
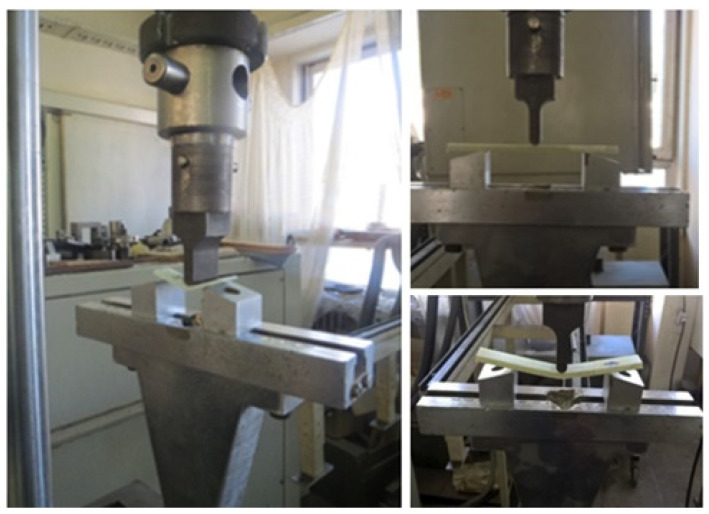
Flexural strength test using a three-point flexural method and the universal testing machine (UTM).

The flexural properties of cut composite samples from the manufacture plates (L-I, L-II, L-III, L-IV, and L-V) were determined with the help of the three-point bending test in accordance with the procedure described in the standard EN ISO 14125 [47]. For that purpose, the computer-controlled universal testing machine (UTM) Hydraulic press, SCHENCK-Hidrauls PSB with a maximal load of 250 kN, constant crosshead speed of 5 mm/min, and span-to-depth ratio of 16:1 was used. The standard dimensions of the tested samples according to EN ISO 14125 are $\left(b\times l\times h\right)mm,$ i.e., (15 × 60 × sample thickness) mm. The rectangular samples were cut in two orientations, the machine direction (MD) and the cross direction (CD), and were tested under the same conditions to ensure reproducibility. The dimensions (length, width, and thickness) of each specimen were measured with the help of a micrometer instrument. The flexural strength of the prepared composite specimens was evaluated using a Universal Testing Machine (UTM), which is illustrated in Figure 4. Load and displacement were recorded by an automatic data acquisition system for each sample. A minimum of five reproducible tests were performed for each sample at room temperature.

The flexural strength $\sigma_{f},$ flexural modulus of elasticity ($E_{f}$), and flexural strain ($\varepsilon_{f}$) of the composite samples were calculated using Equations (1)–(3).

(1) $$\sigma_{f}=\frac{3FL}{2bh^{2}}$$

where

F—load applied to the specimen (N);

b—width of the specimen (mm);

h—thickness of the specimen (mm);

L—length of the span between the supports (mm).

(2) $$E_{f}=\frac{L^{3}}{4bh^{3}}\left(\frac{∆F}{∆s}\right)$$

(3) $$\varepsilon_{f}=\frac{6sh}{L^{2}}$$

where

$\frac{∆F}{∆s}$—slope of the load (∆F) versus deflection (∆s) curve, which represents the rate of change in the load with respect to deflection (N/mm);

s—maximum deflection of the specimen at the center (mm).

### 2.5. Differential Scanning Calorimeters (DSCs) and Tests

In order to conclude that the degree of polymerization of the epoxy resin system was complete in all the samples, a DSC analysis of all composite plates was performed. A sample in a powder form was taken from each plate, and DSC analyses were performed on the DSC Q2000 (Mettler-Toledo TA Instruments, New Castle, DE, USA) with a rate of 40 K/min, in accordance with the procedure described in the standard ISO 11357-1 [48]. For transitions such as the glass transition, DSC enables the measurement of temperatures and heat flows associated with thermal transitions in a material.

### 2.6. Optical and Scanning Electron Microscopy

**Figure 5:**
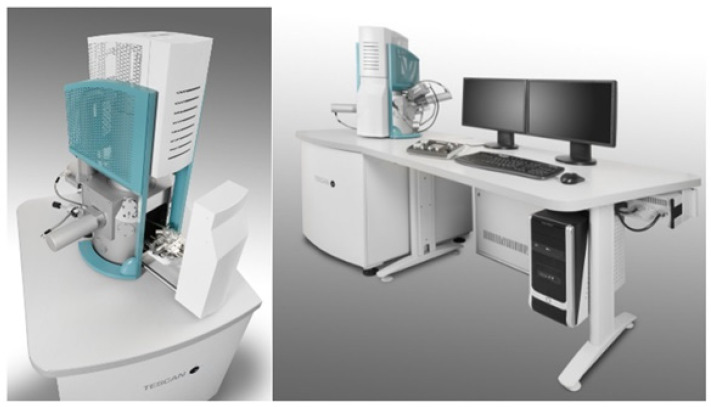
Scanning electron microscope VEGA3 LMU from the company Tescan.

The fracture surface of the composites was studied using a binocular microscope ZOOM 645, Infitek Co., Ltd. Hong Kong Cooperation Zone, Shenzhen, China and a scanning electron microscope type VEGA3 LMU from Tescan, Brno–Kohoutovice, Czech Republic at the laboratories of Goce Delcev University in Stip (Figure 5). For the SEM analyses, the fracture surfaces of the investigated samples were sputter-coated with gold.

## 3. Results and Discussion

### 3.1. Structural and Mechanical Characteristics of E-Glass Woven Fabrics

**Figure 6:**
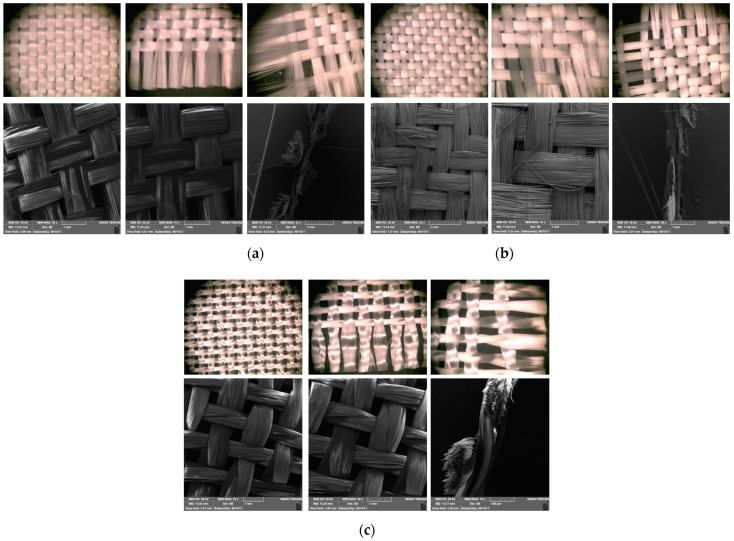
Microphotographs from (**a**) Sample I, (**b**) Sample II, and (**c**) Sample III.

The electron micrographs presented in Figure 6 for test Samples I, II, and III at different magnifications reveal distinct differences in the interweaving of warp and weft yarns in the woven glass fabrics. In the first and second analyzed samples, two diverse types of weaves (plain and twill 2/2 Z) are represented, which belong to the group of basic types of interlacing, while in the third sample, we find the basket 2/2 weave structure (interlacing derived from plain weave).

From the micrographs of Sample I, it is clear that the interlacing of warp and weft yarns is in a 1:1 ratio, where each warp yarn passes over one weft yarn and then under the next, creating a stable and balanced structure. This weave structure provides a firm and strong fabric with a uniform appearance of the fabric. The interlocking nature of the plain weave provides good dimensional stability and minimizes fabric distortion during handling and processing. The tight weave structure offers a relatively smooth surface, which is important for ensuring a good bond between the fabric and the resin in composite applications. However, it has limitations in terms of conformability, resin absorption, and flexibility, which can affect its suitability for certain high-performance or complex composite applications. Due to its structure, plain weave fabrics can be less flexible and harder to conform to complex shapes or curved surfaces compared to other weaves like twill or satin. This can result in wrinkles or gaps when trying to mold the fabric into intricate designs. The tight, closely packed nature of the plain weave results in lower porosity, which can limit the resin flow and wet-out. This may lead to issues in achieving a complete and uniform impregnation of the fabric during composite fabrication, potentially creating voids or weak areas [49]. The electron micrographs for Sample II, represent a 2/2 Z twill weave for which there is the characteristic connection of the warp and weft yarns, whereby Z diagonals are formed on the face of the fabric. In this structure, each warp yarn passes over two weft yarns and then under two, with the interlacing steps shifting by one yarn in each successive row, creating the diagonal pattern. The 2/2 twill weave offers a fabric with good drapability, and there is a certain level of flexibility due to the fewer interlacing points compared to a plain weave. The diagonal structure also makes twill fabrics more resistant to wrinkles and abrasion. This type of weave is particularly advantageous for complex-shaped molds or curved surfaces, as the fabric can easily conform to intricate contours without wrinkling or bunching. The looser structure of twill weave (compared to plain weave) allows for better resin flow and impregnation.

The micrograph of the basket 2/2 weave is represented in Figure 6c, and it is noticed that two warp threads pass together over two weft threads in a repeating pattern, giving a structure similar to a plain weave but with a more relaxed, bulkier texture. It is sometimes called a “double plain weave” due to its interlacing pattern. In this type of weave, the empty spaces between the warps and weft yarns are clearly visible, and this porosity will result in better soaking and infiltration of the resin system into the fabric itself.

The structural characteristics of the applied E-glass woven fabrics were determined using the standard methods ASTM D3776 and BS EN 1049-2 [50,51]. The analysis shows that each type of E-glass woven fabric has a higher density in the warp direction, with a greater number of yarns per centimeter compared to the weft direction. Both the plain (Sample I) and twill (Sample II) weaves have similar warp densities (8 ± 1 ends/cm), whereas the basket (Sample III) weave has a slightly lower warp density (7 ± 1 ends/cm). This reduced density in the basket weave may be attributed to its distinct interlacing pattern, which is generally looser than that of plain and twill weaves. Sample II has a slightly higher weft count (7 ± 1 ends/cm) compared to the other two weaves (plain and basket), both of which have a weft count of 6 ± 1 ends/cm. This difference in weft count may influence the fabric’s flexibility and drapability, whereby Sample II potentially offers better pliability due to its higher weft count. These structural variations impact mechanical properties, as plain and twill weaves, with their denser and more uniform configurations, typically provide higher strength and stability. In contrast, the basket weave’s lower warp density and looser structure might offer increased flexibility but with the potential of reducing its strength.

**Figure 7:**
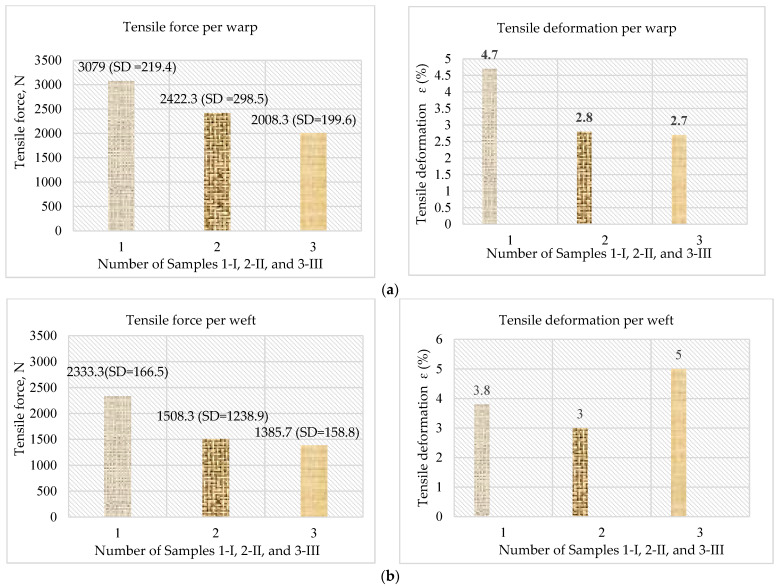
Tensile strength and tensile deformation of applied woven fabric according to (**a**) warp and (**b**) weft.

Figure 7 presents the obtained values for tensile strength (N) and tensile deformation (%) of the analyzed woven fabrics, determined according to the ASTM D 5035 standard [42]. The results are the mean values of three measurements of the tensile strength in the warp (longitudinal) direction and the weft (transverse) direction.

From the obtained results (Figure 7), it can be concluded that the E-glass fabric in-plain interweaving (Sample I) has the highest resistance to tearing forces by warp and weft (3079 N and 2333.3 N). This is also understandable because this type of weave is characterized by the strongest interconnection of the warp and weft yarns. The tensile deformation in the warp direction (4.7%) is moderate, indicating that while the fabric is strong, it is not highly flexible under tension. The tensile strength of the twill fabric (Sample II) in the longitudinal direction is about 20% lower than the tensile strength of the plain fabric but about 18% higher than the tensile strength of the basket fabric (Sample III). Also, the twill fabric is distinguished by a lower tensile strength in the transverse direction (by about 35%) compared to the tensile strength of the plain-woven fabric (Sample I). The tensile deformation in both directions (2.8% and 3%) is lower as well, indicating that the material is stiffer and less flexible under tension compared to the plain weave. Twill’s diagonal structure might improve its ability to distribute stress but results in a somewhat less efficient load-bearing capacity compared to plain weave. Sample III in the basket weave shows the lowest tensile strength in both directions (2008.3 N longitudinal, 1385.7 N transverse), likely due to the weave’s inherent structure. The basket weave, known for its balanced but more open structure, allows for larger interspaces between the yarns, which reduces the compactness and, consequently, the tensile resistance compared to denser weaves. The deformation in the warp direction (2.7%) is also relatively low, suggesting that while the material is somewhat strong, it does not elongate significantly before failure. However, the tensile deformation in the weft direction (5.0%) is the highest, indicating that the fabric is more flexible and can elongate before breaking, which is typical for basket weaves that offer more flexibility. The plain weave (Sample I) shows the highest tensile strength in the warp direction, followed by the twill weave (Sample II), and the basket weave (Sample III) has the lowest. This trend suggests that the warp count plays a significant role in strength, and the plain weave’s structure provides the most resistance to tensile forces. The tensile strength in the weft direction shows a similar pattern, with the plain weave being the strongest, followed by the twill, and the basket weave showing the weakest strength.

Based on the obtained results, it can be concluded that all the analyzed woven fabrics exhibit anisotropic properties in terms of tensile strength, with a higher tensile strength in the longitudinal (warp) direction than in the transverse (weft) direction. The plain weave shows the highest tensile deformation (4.7%) in the warp direction, indicating it is more capable of stretching before failure. The twill and basket weaves have lower deformation, suggesting they are stiffer under tension, with the basket weave showing the least deformation. Interestingly, the basket weave has the highest deformation in the weft direction (5.0%), suggesting it is more flexible compared to the other weaves. The plain and twill weaves are less flexible in the weft direction (3.8% and 3%).

Nonwovens as a textile structure are made from a collection of asymmetrically ordered fibers or chopped yarns that have been stiffened by thermal, chemical, or mechanical bonding techniques. The composition and structure of nonwoven textiles determine their mechanical and structural qualities. The distinctive characteristics of a nonwoven are determined by the type of bonding, the type of fiber, and the manufacturing conditions; this is a different area of study. This textile structure is not a weave, which is characterized by some interconnection of the warp and weft yarns, and because of that, we did not perform structural and mechanical testing. However, the fiber composition and any chemical binders, fillers, or finishes that are applied to or in between the fabric’s fibers determine the structural and mechanical behavior of nonwoven textile constructions. In this research, we only examined the structural and mechanical characteristics of the woven textile constructions.

This study examined the effects of the UD prepreg, woven prepreg, and nonwoven prepreg on the mechanical and physical characteristics of composite plates.

### 3.2. Constituent Content and Void Content of the Composite Plates

The constituent content is crucial for evaluating the quality of the fabricated material. The results from Table 5 show that Samples I to III demonstrate a balanced composition between glass woven fabrics and epoxy resin, with a reinforcing component mass ratio ranging from 67% to 70% and an epoxy resin content from 30% to 33%. This ratio is promising, as a higher glass fabric content contributes significantly to mechanical strength, stiffness, and overall structural performance. Meanwhile, the resin percentage is sufficient to ensure effective bonding, stability, and load transfer within the matrix.

The actual density of the composite was determined on the basis of Archimedes principle. The difference between the theoretical density and actual density was found up to a maximum value 5.28%, which represents the amount of void content present in the developed composites, as shown in Table 5. The suitability of the processing method can be justified with the help of the low void percentage present in the composites.

The fiber volume fraction, or the percentage of the fiber reinforcement into the composite, also affects the mechanical performance of the composite. Composites with higher fiber volume fractions tend to be stronger and stiffer, but as the fiber content increases, processability and cost may become more difficult. According to standard ASTM D 2584 [52], the constituent content of the composite plates was determined. Using the resin weight percentage and reinforcement weight percentage data found in the composite, we calculated the theoretical density of a composite as follows:

$$T_{d}=\frac{100}{\left(\frac{W_{m}}{q_{m}}+\frac{W_{f}}{q_{f}}\right)}$$

where

$T_{d}$ = theoretical composite density;

$W_{m}$ = resin in composite, weight %;

$q_{m}$= density of resin;

$W_{f}$ = reinforcement in composite, weight %;

$q_{f}$ = density of reinforcement.

The Archimedes principle was used to determine the measured composite density in accordance with the ASTM D792 and ASTM D2734 standards [44,45]. The standard difference between the theoretical and measured composite density was used to determine the void content of the composite plates:

$$V\left(\%\right)=\frac{100\;\left(T_{d}-M_{d}\right)}{T_{d}}$$

where

V = void content, volume %;

$T_{d}$ = theoretical composite density;

$M_{d}$ = measured composite density.

The constituent content and void content of the composite plates (L-I, L-II, L-III, L-IV, and L-V) are given in Table 5.

When assessing the quality of the manufactured material, the constituent content is essential. With a reinforcing component mass ratio ranging from 60.7% to 70% and an epoxy resin content of 30% to 39%, Samples L-I through L-IV exhibit a balanced composition between glass woven fabrics and epoxy resin, according to the results in Table 5. This ratio is encouraging because mechanical strength, stiffness, and overall structural performance are all much enhanced by a larger glass fabric percentage. In the meantime, there is enough resin in the matrix to guarantee good bonding, stability, and load transfer. The mass ratio of the reinforcing component in composite samples reinforced with the basket fabric is, in fact, the smallest at 67% due to the open structure of the fabric. Because of the wider inter-yarn spacing in the basket weave, more resin can infiltrate, increasing the resin concentration in comparison to the reinforcing fibers. The unidirectional (UD) reinforced composite samples (L-V) have a composition that is well-balanced. The UD structure has a remarkably high mass ratio of 76% for the reinforcing component and 23% for epoxy resin. Most likely, the fiber content in Sample V results from the resin leaking out in only one direction during the compression process, allowing the resin to flow. As the distance between the two yarn systems increases, the overall mass percentage of the reinforcing component decreases [53,54,55,56,57,58,59]. In this instance, the decreased load-bearing fiber content may result in a loss in mechanical qualities such as flexural strength (Section 3.5 in this publication). Given that denser weaves typically offer higher fiber content and, as a result, stronger mechanical qualities, this realization emphasizes the significance of choosing the right weave structure in composite construction. The mat-reinforced samples had the highest void percentage (5.28%), while the basket fabric-reinforced samples had the second highest proportion (0.85%). With the exception of Samples L-IV, nearly all of these slabs met the criterion because the permissible percentage of voids in them was up to 3%.

### 3.3. Thermal Analysis (DSC) of the Composite Plates

Two factors may contribute to the accumulation of residual stress during the curing process of composite production: matrix and fiber shrinkage because of post-curing temperature reductions or contraction of matrix resin as a result of cross-link polymerization during curing. Damage initiation and progression are impacted by this residual stress, which ultimately leads to composite failure [55,56,57,58,59]. The mechanical performance of the final composite is also influenced by temperature and, thus, the degree of cure distributions [60]. That is why a thermal analysis of all the produced plates (L-I, L-II, L-III, L-IV, and L-V) was performed to see how the curing process was completed.

**Figure 8:**
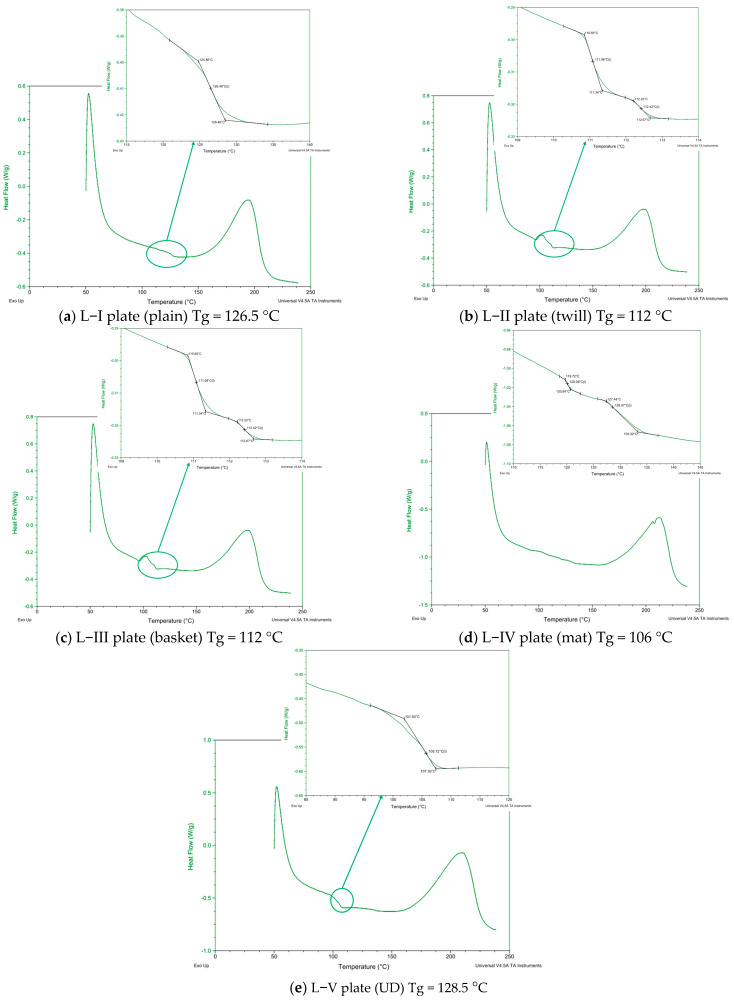
Diagrams from the DSC instrument from which Tg is read for all plates. (**a**) L−I; (**b**) L−II: (**c**) L−III; (**d**) L−IV; and (**e**) L−V.

Samples (in powder form) were extracted from each plate for DSC examination. The testing was conducted using TA DSC equipment that has a heating rate of 40 K/min. The curves obtained from the testing are shown in Figure 8.

The polymerization is complete, according to the diagrams, in all samples, and the glass transition temperature range is from 106 °C to 128 °C. Compared to the other samples (L-I, L-II, L-III, and L-IV), where the curing temperature and time were the same, Sample L-V has a higher Tg value because we used a higher curing temperature. The matrix degrades at temperatures higher than Tg, losing its mechanical qualities and sometimes failing entirely. In our instance, the processing temperature was within the proper range; for curing and post-curing, we used temperatures of 80 °C and 110 °C. The DSC analysis showed that the curing and post-curing process temperatures for composite production result in complete cross-linked polymerization of the polymer matrix in all the composite samples. Therefore, that has no effect on whether the mechanical qualities grow or decrease. The textile structures in our study determine the mechanical characteristics of the textile–polymer composite fabrics.

### 3.4. Static Mechanical Analysis—Flexural Strength and Modulus

**Figure 9:**
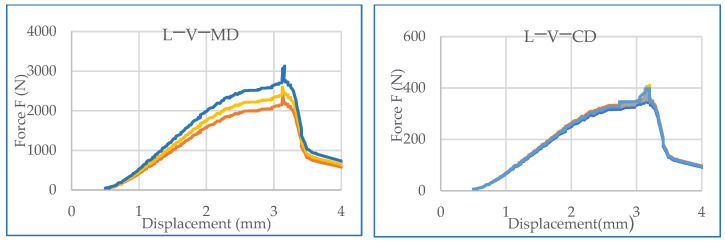
Force and displacement graphs for L−V MD and L−V CD test samples with three replications presented with different colors of the curves

Figure 9 displays the force–displacement curves for a few of the tested samples (Samples L-V) that were acquired from the universal testing equipment. For the other tested samples, the resulting curves have the same look, i.e., they increase linearly until they reach a maximum endurance strength, after which they decline until the test sample is completely destroyed.

**Figure 10:**
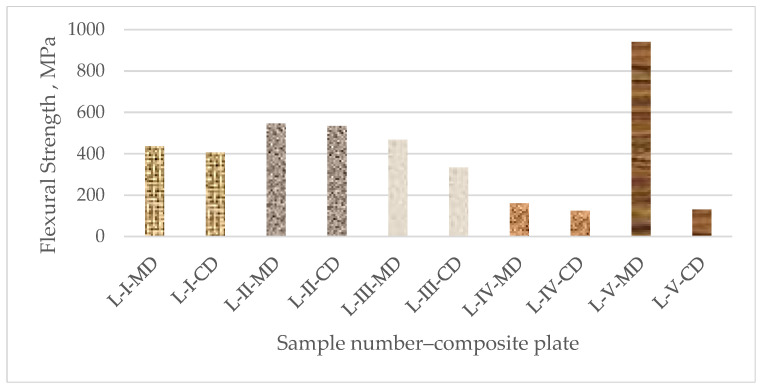
Flexural strength of composite plates.

**Figure 11:**
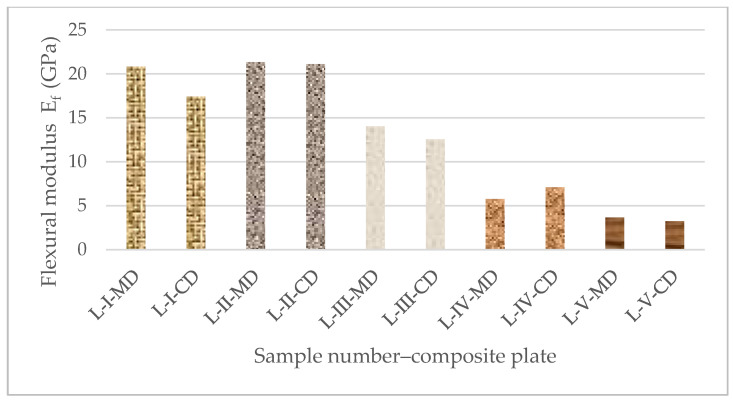
Flexural modulus of composite plates.

Every curve achieves a maximum force known as the maximum endurance strength, which shows the highest load the material can bear before failing. When the maximum load is reached, the curves begin to decline, signifying a reduction in load-carrying ability. This process continues until the sample failure entirely. The flexural strength ($\sigma_{f}$), flexural modulus ($E_{f}$), and flexural strain ($\varepsilon_{f}$) for each group of composite plates were determined from the force–displacement curves with the use of Equations (1)–(3) (see Section 2.4 of this paper). The overall results (average value from three specimens for each of the five types of composite samples) for the flexural properties of all the tested composite samples are presented in Table 6. For an improved analysis and comparison, the flexural strength and modulus for all the composite samples in both directions are also displayed in Figure 10 and Figure 11.

The composite samples reinforced with unidirectional prepregs (L-V) have the highest resistance to bending forces in the longitudinal direction (MD) and the lowest in the transversal direction (CD), according to a comparative analysis of the results obtained for the flexural properties of all sample composite plates. Namely, the test samples L-V-MD have about ten times higher flexural strength than the same sample tested in the transversal direction (L-V-CD). Because of the UD structure, the mechanical characteristics of these samples are unbalanced between the fiber direction and the transverse direction. The UD prepreg has advantages because of its non-weaving process, and for their application into composites, there is a possibility of utilizing the high load-bearing capacity of the fibers when they are loaded in their direction.

However, the woven prepregs (plain, twill, and basket) in the composite laminates L-I, L-II, and L-III exhibit balanced characteristics in both directions. Additionally, those samples exhibit comparable behavior under bending force loads. Although the composite samples reinforced with plain and basket fabrics have comparable flexural strengths, Samples L-II have a flexural strength that is almost 20% higher than them. Nonwoven prepreg-reinforced composite samples (L-IV) exhibit the least resistance to bending pressures in both the longitudinal and transverse directions. Since mat reinforcements usually consist of randomly oriented fibers, which can result in lower directional strength compared to unidirectional or woven fibers, this reduced stress could be an indication of structural differences. The flexural properties of the composites from nonwoven fabrics are strongly influenced by fiber content and fabric direction.

Woven fabric-reinforced composite structures offer certain advantages over conventional unidirectional laminates as well, particularly in applications where structural performance and complex shapes are critical. Using fabric-reinforced composites, thinner laminates and structures with complex geometry can be produced. These composites also have higher impact resistance, stiffness, and dimensional stability compared to unidirectional laminates [61,62]. Their adaptability to complex geometries also makes them a preferred choice in industries like aerospace and automotive, where optimizing material efficiency and structural integrity is essential.

During flexural testing, the outer layers of the composites endure greater loads compared to the middle part. Thus, the flexural strength and modulus are determined by the properties of these outer layers. Under flexural loading, one side of the composite faces the tensile effect while the other side undergoes a compressive load [63]. Zhang [62] investigated the effect of the tensile–compression direction by applying flexural testing on unsymmetrical composites from two opposite sides. The asymmetric composites consist of a glass fiber layer on one side and a carbon fiber layer on the other side. In the case of composite asymmetry, the axis of flexural bending can deviate from the material’s mid-plane, contrary to the general assumption; thus, suitable modeling corrections should be considered [64].

The typical load–displacement graph of textile-reinforced polymer composites includes a broad plateau following the load drop ending the linear elastic region. The large plateau forms evidence for the remarkable damage tolerance of these structures [43]. While matrix cracking is thought to have little effect on the flexural strength, the effect of matrix cracking on the fiber stress distribution is not yet fully understood [65]. The place and the mode of damage onset are strongly influenced by the fabric structure and geometry as well as composite layer sequencing/stacking [64,65]. Composites exhibit complicated failure modes depending on a number of factors, including load behavior and direction, fabrication parameters, and composite structure. Fracture of fibers, matrix fracture, interfacial debonding, and delamination are among the failure modes that the composites show [60,61].

When discussing the mechanisms behind the thermal and mechanical properties of composites, particularly those made with textile fibers, it is important to understand how both the fiber reinforcement and the matrix material contribute to the overall behavior. The properties of these composite materials are highly influenced by the interaction between the fibers and the matrix, the characteristics of the fiber itself (e.g., stiffness, strength, and thermal properties), and the specific processing conditions.

A strong bond between the fiber and matrix prevents relative motion between layers and delays crack propagation. Additionally, the fiber orientation plays a significant role; composites with fibers aligned in the direction of loading tend to have higher resistance.

Composites subjected to bending or shear forces depend on the ability of the matrix to resist sliding between fiber layers. The orientation of fibers (e.g., unidirectional, bidirectional, woven fabrics) influences the flexural/shear strength. In woven or braided fabrics, the fibers resist bending in multiple directions, providing better overall flexural strength. A strong fiber–matrix bond ensures that stress is efficiently transferred from the matrix to the fibers, thereby improving the flexural strength. If the matrix fails to bond well with the fibers, the load transfer is less effective, and the composite’s strength is compromised. The strength of the fiber–matrix interface is a key determinant of the flexural strength. A weak interface leads to delamination, which significantly reduces the flexural strength. In contrast, a strong interface ensures better load transfer and enhanced flexural and shear resistance.

### 3.5. Optical and Scanning Electron Images of Prepregs and Composite Plates

**Figure 12:**
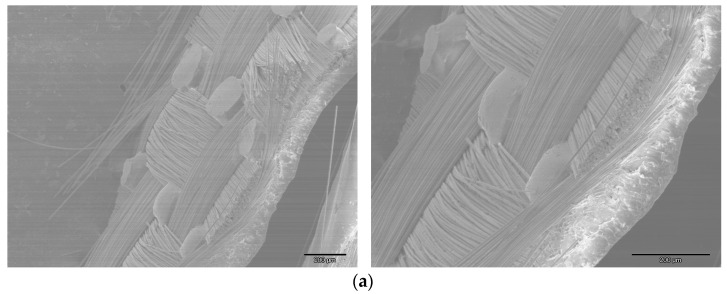

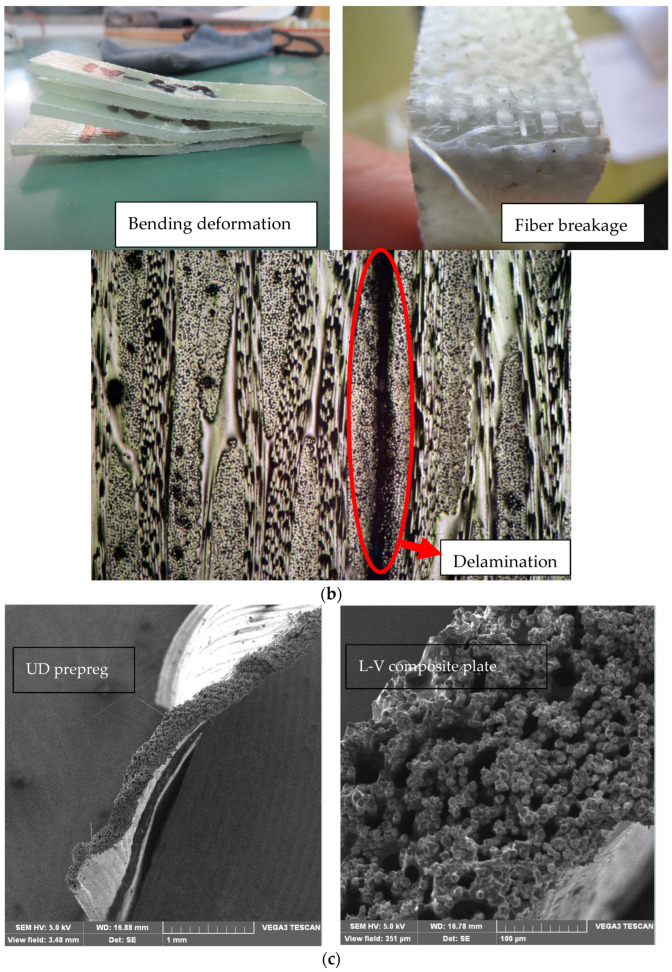
Optical and scanning electron images of prepregs and composite plates. (**a**) SEM image of glass woven prepregs: good fiber–matrix interface in the prepreg (in the impregnation process). (**b**) Illustrations of the deformation and fiber breakage after the breakage test of Sample L-I-MD and an optical microscope image. (**c**) SEM image of the glass UD prepreg (**left**) and SEM image of a cross-section of the broken L-V composite plate (**right**) after testing.

Mechanical behavior is also significantly influenced by the matrix material. For the load to be transferred from the matrix to the fibers effectively, the matrix and textile reinforcement must form an effective bond. Several SEM pictures were captured to determine whether the reinforcements and resin in the composites had a good interaction. Almost all composite samples have a pleasant interface thanks to soaking or the impregnation of the fibers, as seen in the various photos in Figure 12.

During the impregnation process and the creation of the permanent prepreg, the image (Figure 12a) demonstrates good resin impregnation of the glass fabric (woven) fibers. The textile reinforcement and its interactions with the matrix material play a major role in the failure mechanisms of polymer composites. A common failure mode is delamination, which occurs when inadequate interlaminar bonding causes layers of composite material to separate. Fiber interlacing in woven and braided fabrics strengthens the link between layers, resulting in improved resistance to delamination (Figure 12b). When the load is greater than the fibers’ tensile strength, fiber rupture is the predominant failure mode in unidirectional composites. With woven fabrics, this is less of an issue because the load is distributed throughout the warp and weft fibers (Figure 12b). Because of the good interface between the fibers and epoxy resin, as well as the fact that the load is dispersed across the warp and weft threads, woven textiles have a better resistance to delamination, according to the microphotographs taken from the composite plates (after they ruptured during mechanical testing). However, in unidirectional composites, fiber rupture is the most common failure mode when the load exceeds the tensile strength of the fibers. These SEM analyses are consistent with the findings on the flexural characteristics of suitable composites.

## 4. Conclusions

The results of this study show that the mechanical characteristics of composite materials are greatly impacted by the fiber orientation in the textile structure as reinforcement. In terms of tensile strength for the tested E-glass woven fabrics (plain, twill, and basket), the analysis shows that the structure of the plain weave offers the greatest resistance to tensile pressures and that the warp count has a major impact on strength. The plain weave is the strongest, followed by the twill, and the basket weave is the weakest, according to a similar pattern of tensile strength in the weft direction.

In terms of the mechanical behavior of the composite samples, those reinforced with unidirectional reinforcement have a particularly high load-bearing capacity in the longitudinal direction and the strongest resistance to bending forces in that direction. Although the composite samples reinforced with twill textiles show higher flexural strengths comparable to those reinforced with plain and basket fabrics, the composites reinforced with woven textile structures (plain, twill, and basket) exhibit balanced properties in both directions. When compared to the composites reinforced with woven or unidirectional textiles, the nonwoven-reinforced composites with randomly oriented fibers have lower flexural strengths.

Polymer–textile composites have a wide range of possible uses, from the automotive and aerospace sectors to sports gear, medical products, etc. Applications where the load is delivered mostly in one direction, such as beams, stiffeners, or other structural components in automotive and aerospace applications, are best suited for unidirectional reinforcement. In complex load-bearing components, where the composite must sustain loads from several directions, woven reinforcing is advantageous. For non-structural automotive components, nonwoven reinforced composites are frequently utilized. Nonwovens are utilized in geotextiles, filtering, medicine, and acoustic purposes.

As research and development in the field of polymer–textile composites continue to advance, we can expect to see even more innovative applications in the future. The current research aims to highlight the latest advancements in polymer–textile composites and their potential impact on various industries in terms of enhancing material properties and exploring new applications.

## Figures and Tables

**Table 1:** Basic characteristics and assignation of textile structures.

Characteristic		Assignation			
		Woven			Nonwoven
		Sample I	Sample II	Sample III	Sample IV
Type of textile structure		Plain 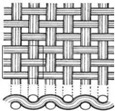	Twill 2 × 2 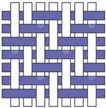	Basket 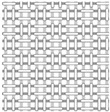	Mat 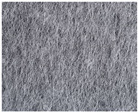
Reference		EW300-2000	T-25/1-76	EWB350-2000	EasyMat
Supplier/Producer		Sinoma Science & Technology Co., Ltd., Beijing, China			FRP Services & Co., Aix-en-Provence Cédex3, France
Fiber type		E-glass	E-glass	E-glass	E-glass
Mass per unit area (g/m^2^)		300 ± 20	320 ± 20	320 ± 25	300 ± 25
Thickness (mm)		0.3	0.32 ± 0.05	0.31	0.3
Width (cm)		100	92	100	125
Count (ends/cm)	Warp	8 ± 1	8 ± 1	6 ± 1	Moisture content (%) <30
	Weft	7 ± 1	6 ± 1	5 ± 1	
Strength (N/25 mm)	Warp	≥2000	≥2000	≥1800	
	Weft	≥1200	≥1400	≥1200	

**Table 2:** Characteristics of the components of the epoxy resin system.

Epoxy Resin (D.E.R 3821)		Polypox H 766	
Epoxide equiv. weight (g/eq)	176–183	H—equivalent weight (g/Equiv.)	55
Epoxide percentage (%)	23.5–24.4	/	/
Epoxide group content (mmol/kg)	5460–5680	Amine number (mg KOH/g)	540 ± 15
Color (Platinum cobalt)	125 Max.	Color (Gardner)	blue
Viscosity @ 25 °C (mPa∙s)	9000–10,500	Viscosity at 25 °C, (mPa∙s)	14
Density at 25 °C, (g/cm^3^)	1.16	Density at 25 °C, (g/cm^3^)	0.94 ± 0.05
Epichlorohydrin content (ppm)	5 Max.	/	/

**Table 3:** Characteristics of prepregs.

	Unit	Produced Prepregs (Woven/Nonwoven)	Commercial UD Prepreg (SIGRAPREG^®^ G U300-0/NF-E320/35%)
Fiber type	/	E-glass	E-glass
Volatile content	% wt.	Less than 2%	Less than 1%
Resin content	% wt.	30–33% (+/−5%)	35% (+/−2%)
Prepreg areal weight	gr/m^2^	500	462
Fiber areal weight	gr/m^2^	300–310	300
Thickness	mm	0.5	0.46
Width	mm	920–1000	420

**Table 4:** Assignation of the composite plates and processing conditions.

	Sample L-I	Sample L-II	Sample L-III	Sample L-IV	Sample L-V
Structure of laminate panel	10-layer prepreg with plain fabric	10-layer prepreg with twill fabric	10-layer prepreg with basket fabric	10-layer prepreg with mat	10-layer UD prepreg
Temperature of curing (°C)	80	80	80	80	90
Curing time (min)	60				
Temperature of post-curing (°C)	110	110	110	110	110
Post-curing time (min)	60				
Specific pressure kg/cm^2^ (bar)	14 (30)				

**Table 5:** Constituent content and void content in the composite samples.

Sample Number	Mass Ratio of Resin, $W_{m}$ (%)	Mass Ratio of Reinforcement, $W_{f}$ (%)	Volume of Resin, $V_{m}$ (%)	Volume of Reinforcement, $V_{f}$ (%)	Average Voids (%)
L-I	30.94	69.06	49.74	47.70	2.56
L-II	29.81	70.19	49.08	49.67	1.25
L-III	33.40	66.60	53.59	45.92	0.85
L-IV	39.22	60.78	58.41	38.87	5.28
L-V	23.30	76.10	41.18	56.37	2.46

**Table 6:** Flexural strength, modulus, and strain at all composite plates.

Sample Number		Force $F_{\max}\;(N$)	Flexural Strength $\sigma_{f}$ (MPa)	Flexural Strain $\varepsilon_{f}$ (%)	Flexural Modulus $E_{f}$ (GPa)
L-I	L-I-MD	543.364	436.733 (23.7) *	2.265	20.832
	L-I-CD	506.286	406.544 (17.1) *	3.259	17.420
L-II	L-II-MD	603.63	546.934 (8.2) *	3.726	21.360
	L-II-CD	612.79	534.399 (57.0) *	3.453	21.120
L-III	L-III-MD	904.85	468.808 (24.1) *	3.774	14.045
	L-III-CD	659.18	333.63 (18.8) *	3.767	12.547
L-IV	L-IV-MD	648.70	161.589 (23.6) *	5.081	5.771
	L-IV-CD	647.31	125.053 (26.4) *	3.812	7.101
L-V	L-V-MD	3189.5	940.108 (98.0) *	86.922	3.662
	L-V-CD	472.36	131.3775 (6.0) *	23.938	3.250

* SD—standard deviation.

## Data Availability

Data are contained within the article.. The data presented in this study are available from the corresponding author upon request.
